# The Application of Human iPSCs in Neurological Diseases: From Bench to Bedside

**DOI:** 10.1155/2016/6484713

**Published:** 2016-01-06

**Authors:** Nina Xie, Beisha Tang

**Affiliations:** Department of Neurology, Xiangya Hospital, Central South University, Changsha, Hunan 410008, China

## Abstract

In principle, induced pluripotent stem cells (iPSCs) are generated from somatic cells by reprogramming and gaining the capacity to self-renew indefinitely as well as the ability to differentiate into cells of different lineages. Human iPSCs have absolute advantages over human embryonic stem cells (ESCs) and animal models in disease modeling, drug screening, and cell replacement therapy. Since Takahashi and Yamanaka first described in 2007 that iPSCs can be generated from human adult somatic cells by retroviral transduction of the four transcription factors, Oct3/4, Sox2, Klf4, and c-Myc, disease specific iPSC lines have sprung up worldwide like bamboo shoots after a spring rain, making iPSC one of the hottest and fastest moving topics in modern science. The craze for iPSCs has spread throughout main branches of clinical medicine, covering neurology, hematology, cardiology, endocrinology, hepatology, ophthalmology, and so on. Here in this paper, we will focus on the clinical application of human iPSCs in disease modeling, drug screening, and cell replacement therapy for neurological diseases.

## 1. Introduction

**Figure 1:**
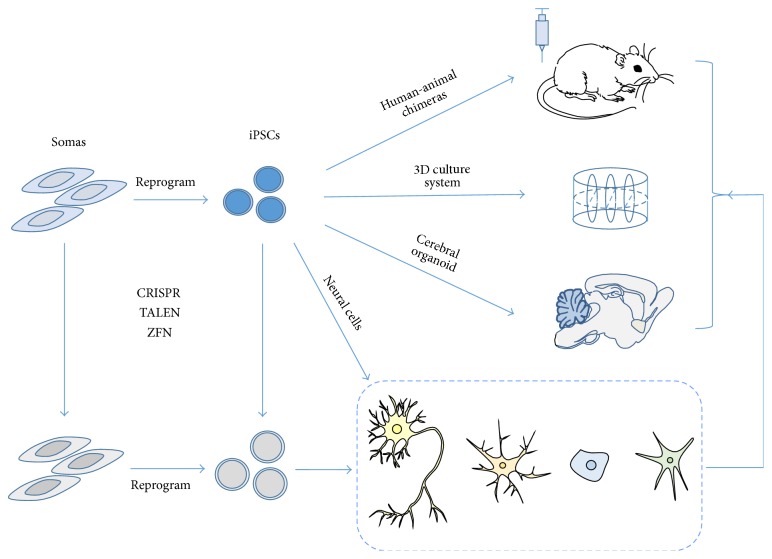
Applications of human iPSCs and iPSC derived neural cells in neurological diseases. In principle, induced pluripotent stem cells (iPSCs) are generated from somatic cells by reprogramming and gaining the capacity to self-renew indefinitely as well as the ability to differentiate into cells of different lineages. Up to date, the main applications of iPSC products in neurological diseases have included disease modeling, drug screening, and cell replacement therapy, for which the successful reprogramming of somatic cells to iPSCs is the most fundamental step. By generating human-animal chimeras, 3D culture systems, and cerebral organoids with disease specific iPSCs and iPSC derived neural cells, researchers can have various systems for disease modeling and drug screening. By inducing disease relevant cell types into wild type (WT) iPSCs, researchers and clinicians can have normal cells for cell replacement therapy. Recently, the rapid development of genome editing technology, including TALEN, ZFN, and CRISPR, makes it possible for diseases with defined genetic disorders to switch phenotype between WT and mutant at the single cell level, providing a new strategy to create syngeneic WT cells for cell replacement therapy [103]. Overall, human iPSCs have a great potential for clinical applications in neurological diseases.

In principle, induced pluripotent stem cells (iPSCs) are generated from human somatic cells by reprogramming and gaining the capacity to self-renew indefinitely as well as the ability to differentiate into cells of different lineages. Human iPSCs have absolute advantages over human embryonic stem cells (ESCs). On the one hand, as an unlimited therapeutic source, human iPSCs overcome the ethical issues faced by human ESCs, for human adult somatic tissue is much more accessible than embryonic tissue both technically and ethically. On the other hand, as patients derived stem cells, human iPSCs can better recapitulate disease phenotype and pathological process without having the interspecies difference existing in animal models. Since Takahashi and Yamanaka first described in 2007 that iPSCs can be generated from adult somatic cells by retroviral transduction of the four transcription factors Oct3/4, Sox2, Klf, Klf4, and c-Myc [1, 2], disease specific human iPSCs lines have sprung up worldwide like bamboo shoots after a spring rain, making iPSCs one of the hottest and fastest moving fields in modern science. The craze for iPSCs has spread throughout main branches of clinical medicine, covering neurology [3–6], hematology [7, 8], cardiology [9, 10], endocrinology [11, 12], hepatology [13], ophthalmology [14, 15], and so forth. Here we will focus on the clinical applications of human iPSCs in neurological diseases' modeling, drug screening, and cell replacement therapy (Figure 1).

## 2. Overall Disease Modeling Strategies and Challenges

The first strategy would be reprogramming patients somatic cells directly to iPSCs. As long as there is accessible patient somatic tissue, a disease specific iPSC line can be gained using suitable reprogramming methods, whether it is a neurogenetic disorder with defined genetic reasons or a sporadic disease with no defined genetic reasons. Up to date, neurological disease models using this strategy have included fragile X syndrome (FXS) [6], Rett syndrome [16], Down syndrome [17], Parkinson's disease (PD) [4, 18–22], Alzheimer's disease [5, 23–27], and schizophrenia (SCZH) [28, 29]. Significant progresses have been made by using this iPSC based strategy although it is at a very early stage. For example, some general neurological disease phenotypes have been revealed, such as synaptic deficiency, inadequate neuronal maturation, abnormal response to oxidative stressors, and mitochondrial deficits, which would provide insight into new therapy targets or drug screening strategies [19–21, 23, 28, 30–33]. However, as promising and straightforward as it is, this strategy is also facing lots of challenges. First, the iPSC model is not an equal substitute for the ESC model. For example, studies of FXS-ESCs showed that FMR1 is unmethylated and expressed at a level close to the normal [34]. By contrast, FMR1 in iPSCs reprogrammed from somatic cells of FXS patients remains silent [6]. To elucidate this contradiction, further biological studies focusing on the difference between ESC and iPSC are needed. It is very likely that some key pathogenic mechanisms are hiding behind the subtle difference between iPSCs and ESCs. Another challenge is the variability between different patients or different clones derived from the same patient. For some diseases like SCZH, it is hard to determine whether the observed phenotype is really disease relevant or merely due to specific genetic background. Therefore, instead of just comparing one single iPSC line from one patient versus one control, it is indispensable to generate a panel of disease relevant iPSC lines and controls from different patients and normal people to overcome the bias attributed to the individual iPSC line variation. Last but not least, there are still lots of technical limitations to model the late-onset disease like PD. The manifestation of PD associated phenotypes requires environmental stimuli, such as progerin, to accelerate the pathological process. Thus, it would be of great help to define age-related markers which could be used to monitor the induced aging process to mimic the decade long aging process in several months [35].

The second strategy would be generating humanized animals with human iPSCs via neonatal mouse brain injection [36] and human mouse chimera [37, 38], which is a technologically conventional but ethically controversial strategy. It has been reported that undifferentiated human ESCs directly injected into the brain ventricles of fetal mice can integrate into the mice brain and form human neurons and glia [39]. Similar studies further support that stem cell injection, either human ESCs or human ESC/iPSC derivatives, has a great potential in cell replacement therapy [40–42]. For example, It has been reported that locomotor recovery was associated with engraftments of human neural progenitor cells (NPC) in the spinal cord-injured immunodeficient mice [42]. Albeit the underlying mechanisms for these phenomena remain mysterious, this modeling strategy is still the basis for studies involving cell replacement therapy. Of note, much less evidence has shown to generate humanized animals with direct iPSCs injection[43], which is probably due to the difficulty controlling in vivo differentiation of iPSCs precisely, suggesting iPSC derived NPC would be a better choice for cell replacement therapy.

The 3rd strategy would be establishing three-dimensional (3D) structured in vitro models that can emulate human homeostasis vividly. At present two-dimensional (2D) culture systems are the platform most often being used. However, these 2D systems cannot mimic the delicately structured in vivo environment. Instead, 3D culture system could emulate the complex in vivo environment in vitro by mimicking the highly organized cytoarchitectural features. First, the 3D culture system can achieve compartmentalization of different cell types. Different cells will reside in organized chambers to mimic the architectural features in real tissue niches [44, 45]. In addition, based on compartmentalization of cells, using special biomaterial that can create concentration gradients, researchers can better control delivery of biochemical compounds towards specific cells types, which mimics the delivery of signal cues in vivo. For instance, by utilizing a 3D micropatterned culture system, researchers could regulate synapse distribution via concentration gradients of neurotrophic factors and cell layer positions. It is observed that synergistic NGF/B27 gradients could increase synaptic density by stimulating growth of cortical neurons, which would be disturbed by homogenous B27 distribution, while cell layer positions could impact the spatial distribution of synapses [46]. Lastly, it should not be ignored that the combination of iPSCs and microfabrication technologies also holds great potential for tissue engineering. Engraftment of human iPSC derived NPC into nanofibrous tubular scaffolds resulting in nerve regeneration is a typical example [47]. In a word, not only can the 3D culture system give us highly organized in vitro models to study but also it enables us to control the cellular microenvironment more precisely.

Last but not least would be the newly developed exciting technology, the mini brain. In 2013, Austrian scientists Lancaster et al. reported successful generation of cerebral organoids, known as the mini brains, in a dish by culturing human iPSC in a fine-tuned 3D culture system. Basically, it comprises multiple discrete, albeit interdependent embryonic brain like regions. Despite lacking delicate organizations and structures that develop in vivo, such as the complete cortical lamination, this model represents a great leap towards modeling the most complex tissue, human brain. On the one hand, the cerebral organoids can reach up to 4 mm in size and can grow in vitro as long as 10 months with a simplified protocol using bioreactor. On the other hand, they are much more closer to embryonic brains compared to those generated via conventional protocols [48]. More importantly, they can recapitulate human cortical development features that mouse models cannot achieve, which is termed “the characteristic progenitor zone organization with abundant outer radial glial stem cells.” Researchers further utilize this method to model a developmental disorder, microcephaly. Interestingly, this human iPSC derived mini brain can mimic severe phenotypes which cannot be manifested in mouse models carrying the same mutation [49]. Overall, it is very promising that in the near future more and more neurological diseases will have corresponding mini brain models to study. And mechanisms remaining mysterious now, especially those involving neurodevelopmental diseases, can be unveiled at that time.

## 3. Drug Screening Strategies and Challenges

Generally, the prevailing pharmaceutical pipeline is inefficient and expensive, whereas more than 90% of the drugs tested in clinical trials fail to be approved [50]. Furthermore, among those hit compounds selected through non-human iPSC platforms, not every candidate is effective on humans, and one of the most important reasons is the lack of faithful disease models. Since patients derived iPSCs can better emulate the real disease mechanisms, it is reasonable to postulate that the patients derived iPSCs based high throughput screens would be a better strategy for drug screening. For example, in the study using human iPSC derived dopaminergic neurons to evaluate candidate PD therapeutic agents, of the 44 hit compounds selected by rodent systems, only 16 were demonstrated therapeutic effects in the human PD model [18], suggesting the superior specificity conferred by the human iPSCs based platform. To establish highly efficient drug screen platforms, two issues are essential: the large-scale production of iPSCs or iPSCs derived neural cells as well as the definition of in vitro readouts suitable for high throughput assays [43]. A good example is a recent drug screening for compounds that can restore FMRP expression in FXS NPC. To adapt related experiments to be done in large scale, researchers first induced FXS iPSC to NPC which is easy manipulating and can rapidly grow in monolayer. Next, FMRP translation level was defined as the readout and a sensitive and quantitative TR-FRET-based FMRP assay was developed, in which FMRP would be detected by a pair of FMRP antibodies, one labeled with a donor dye and the other labeled with an acceptor dye. Briefly, NPC was first seeded into a 1536-well plate, followed by stimulation of chemical libraries comprising 1280 drugs. After that cells were lysed and incubated with the pair of FMRP antibodies. Fluorescence signals given by positive wells with FMRP restoration would be detected and analyzed by the plate reader. In this way, 6 hits were identified and 4 of them were confirmed by secondary qPCR assays [51]. Similar screening platforms have also been applied in other neurological diseases, such as familial dysautonomia [52] and spinal muscular atrophy (SMA) type I [53]. Taken together, this screening strategy is more time saving and less expensive as compared to those traditional ways.

Aside from evaluation of therapeutic effects of a large number of chemical compounds, human iPSC based platforms can also be used to assess cell type specific off-target effects and toxicities. For instance, inducing human iPSCs to hepatocytes and cardiac myocytes provides the opportunity to assess whether compounds of interest have true hepatotoxicity and cardiotoxicity [50]. Moreover, by screening compounds on a cell panel comprising iPSC lines or iPSC derived affected cell types from different patients, compounds that are only effective on certain patients, either with specific genetic profiles or with disease subtypes, can be identified [43]. In this sense, the iPSC drug screening strategy will help narrow target patient populations, contributing to the individualized medicine in the future.

## 4. Cell Replacement Strategies and Challenges

The most exciting potential of human iPSCs would be the cell replacement therapy. The hematopoietic stem cells have already been used in clinic to treat disease such as multiple myeloma and leukemia [54]. Recently, Japanese scientists applied human iPSCs in curing ophthalmological diseases in real patients [55]. It has also been well envisioned that in the near future we can have human iPSC derived organs for transplantation surgery [56]. Human iPSCs and iPSC derivatives have been proven to have therapeutic potentials in neurological diseases including spinal cord injury [42, 57], Huntington disease [58], and PD [59]. However, exciting as these results were, safety issues such as tumor formation and inappropriate localization of transplanted cells were also reported. Therefore, to achieve cell replacement therapy in real clinical settings, strict standards should be set to ensure quality control of clinic grade human iPSCs. Below we will discuss each of them briefly.

### 4.1. Integration-Free Reprogramming Methods

There has been the concern that virus vector mediated reprogramming may introduce unwanted insertion of vector fragments into the iPSC genome, which may affect the biological properties of the derived iPSCs and even induce malignant transformation, rendering the iPSC unsafe for clinic use. To solve this, researchers have been working on integration-free reprogramming methods to get safer iPSCs. A good example is the lentiviral vector plus Cre strategy, in this way, the reprogramming vectors flanking by loxP sites will be excised by Cre-recombinase transfected transiently [4]. But this is labor and time consuming and vector DNA external to the loxP sites may still remain integrated. Other approaches include piggyBac transposon [60, 61], episomal vectors [62], adenoviral vectors [63], sendai vectors [64], protein transfection [65, 66], nucleofection [67], and small molecules delivery [68, 69]. Although they can achieve the integration-free purpose, they share the same limitation which is the extremely low reprogramming efficiency compared to lentiviral vectors. Progress has been made in the significant enhancement of iPSC generation efficiency by Chen and Jin group, in which the transactivation domain of the Yes-associated protein is fused to the defined transcription factors, Oct4, Sox2, Nanog, and Klf4 (OSNK), to establish a new reprogramming system OySyNyK which can initiate rapidly within 24 h with up to 100-fold higher efficiency compared to the conventional OSNK system [70]. Hopefully progress in such studies can finally pave the way for producing the real clean and safe iPSCs efficiently.

### 4.2. Tumorigenicity

Tumorigenicity refers to the ability of iPSCs or iPSC derived cells to form tumors after being transplanted into hosts, which is often evaluated by the teratoma formation propensity assay. Up to date it remains mysterious how teratoma forms in transplantation settings using syngeneic iPSCs or iPSC derivatives. The first reason is that the cell source giving rise to the teratoma is unclear. One possible hypothesis is that undifferentiated iPSCs continually present within the iPSC derived progenitor cells or terminally differentiated tissues are the source of teratoma initiation [71, 72]. Another important reason is that the risk factors affecting teratoma formation are versatile. The first risk factor is the retroviral transgene integration. A major concern is that off-target integration of transgene elements and dysregulation of stem cell programs may cause aberrant tumor related genes expression [73, 74]. Moreover, reactivation or incomplete suppression of retroviral transgenes has been thought to be correlated with increased tumorigenicity. In support, transplantation of secondary neurospheres obtained from a partially reprogrammed iPSC line into the brains of immunodeficient mutant mice results in robust teratoma formation due to the incomplete suppression of transgenes encoding Oct4, Sox2, Klf4, and c-Myc [71]. Optimistically, this concern may be eliminated by using integration-free methods for reprogramming as mentioned above. Another factor that may play a role is the somatic tissue origin. Miura group reported that secondary neurospheres from murine iPSCs reprogrammed from different adult mouse tissues varied substantially in their teratoma-forming propensity. For example, secondary neurospheres derived from iPSCs generated from adult tail tip fibroblasts showed significantly higher tumorigenicity compared to those generated from mouse embryonic fibroblasts [75]. However, this could also be caused by the fact that different tissues have different mutation accumulation, epigenetic memory, and age. More evidence is needed to support this view. If somatic tissue origin really contributes to tumorigenicity, careful selection of original somatic tissue for reprogramming should be considered as a major standard in clinical application, because this will affect what the patient will suffer from at the very beginning, an invasive surgery or just pulling out a single hair. Overall, all evidence shown above is from mice. Since tumor formation mechanism is complex and could be species specific, more evidence from primates or human patients is needed.

Although mechanisms underlying tumorigenicity remain unknown, clinical trials using autologous patient derived iPSCs are already underway marked by the Takahashi group using patient specific iPSC derived retinal pigment epithelium (RPE) cells for the treatment of wet type age related macular degeneration. Notably, in the preclinical study they evaluated the tumorigenicity of patient iPSC derived RPE using immunodeficient rodents. By transplanting iPSC derived RPE cells into 65 nonobese diabetic mice subcutaneously and into the subretinal space of 26 nude rats they concluded that the tumorigenic potential of the patient iPSC derived RPE cells is negligible, for no tumors were found in the following 6–15 months of monitoring [76]. However, it is still risky to state that the iPSC derived RPE cells which are safe for rodents are also safe for human. Thus, strict preclinical evaluation and posttransplantation monitoring should be mandatory to ensure clinical safety. For example, primate models would be a more reliable system compared to rodent models for tumorigenicity assessment [77]. Positive selection of differentiated cells without contamination of undifferentiated cells before the transplantation surgery may also help reduce the risk of tumorigenicity [78]. A more fundamental and ideal solution for the posttransplantation setting would be the prodrug strategy or suicide genes controlled by inducible promoters [79]. By integrating a gene coding for enzymes that can convert prodrug to toxins or a suicide gene into the cells to be transplanted, researchers and clinicians can eliminate the transplanted cells easily just by administrating corresponding drugs when there is tumorigenic event ongoing [80, 81]. However, given all the technical limitations in the way, applying these strategies in real clinical cases still has a long way to go.

### 4.3. Immunogenicity

Another major concern for clinical transplantation of iPSCs is the immunogenicity. Whether syngeneic iPSC is immunogenic remains controversial. The debate was stimulated by a study in 2011 reporting unexpected immune rejections to teratomas derived from syngeneic murine iPSCs. Before that people believed that syngeneic iPSCs or iPSC derived cells are self-tolerant, which is a perfect autologous transplantation source. However, in this study, it is indicated that iPSC derived teratomas have significant higher immune rejection responses than the ESC derived counterparts. The B6 mice recipients receiving syngeneic ESCs transplantation showed efficient teratomas formation with negligible rejection, while those receiving syngeneic iPSCs transplantation, whether reprogrammed from mouse embryonic fibroblasts using retroviral transduction or episomal method, showed robust immune rejection evidence including failure of teratomas formation, T cell infiltration, and tissue damage [73]. In contrast to this study, other two later studies exploring differentiated cells, more therapeutically relevant, showed the opposite results. The first study showed negligible immune rejection of terminally differentiated cells of skin grafts and bone marrow, which are derived from chimeric iPSC or ESC derived mice [82]. The other study observed no immune rejections of terminally differentiated cells from both iPSCs and ESCs representing three germ layers by assessing immunogenicity in vitro and after their transplantation into autologous recipients [83]. Both of the two studies optimistically came to the same conclusion that syngeneic iPSCs or iPSC derivatives are the safe source for cell replacement therapy. Whether such variability between studies is due to the difference between different ESCs and iPSCs lines is unknown yet. Interestingly, a recent study indicated that the differentiation of iPSCs may result in a loss of immunogenicity and the induction of self-tolerant immune response [84], which helped explain the discrepancy of the studies discussed above. Moreover, another recent study of Zhao group reported differential immunogenicity of differentiated cells from syngeneic iPSCs in a humanized mouse model with a functional human immune system, where the iPSC derived smooth muscle cell is highly immunogenic while the iPSC derived retinal epithelial cell is tolerant, though direct transplantation of iPSCs is still immunogenic as their previous results [85]. So far it is difficult to form a hypothesis to explain different phenomena observed by different study groups due to poor understanding of underlying mechanisms. However, the consensus is that improvement in reprogramming technology and insight into the underlying mechanisms is needed for generating clinical grade iPSCs. Similar to tumorigenicity, evaluation of immunogenicity prior to transplantation should also be mandatory.

### 4.4. Promising Preclinical Trials on Animal Models

The first in human clinical trials using stem cell products for neurological diseases is the one launched by Geron in 2009, aiming at remyelinating denuded axons in patients of thoracic level spinal injury with human ESC derived oligodendrocytes which have been preclinically proven to be safe and effective [86]. However, due to financial concerns, this trial was terminated 2 years later [87]. Up to date, no such clinical trials using iPSC products have been reported; however, types of neurological diseases with preclinical data supporting clinical trials using iPSC products are increasing, including PD [88], Huntington's disease [89], amyotrophic lateral sclerosis [90], and SMA [91]. Among them, PD is probably the most promising neurodegenerative disease for cell replacement therapy trial, because it is mainly caused by the loss of midbrain dopaminergic neurons. As a proof of principle, transplantation of midbrain dopamine neurons could be an effective treatment by covering the shortage. Although dopaminergic reagents have been proven to be an effective treatment strategy, it is already recognized that the curative effect of the medications would be compromised due to adverse effects over time. As the logical next step, cell replacement therapy is viewed as a better alternative by scientists, with the potentials of overcoming limitations of medications, such as off-target effect and nonphysiological delivery of dopamine [92]. Clinical trials using fetal brain tissue transplantation and ESC derived neurons for PD have come into reality, highlighted by the “TRANSEURO” and “GForce-PD initiative” programs [92, 93]. However, given the safety issues discussed above, iPSCs and iPSC derivatives based cell therapy are still at the preclinical stage. Cell replacement therapy using iPSC derived neural cells has been trialed on rodent and non-human primate PD models with encouraging outcomes. For example, after transplantation rodent PD models all showed alleviated behavioral phenotypes, such as reduced amphetamine-induced rotations with no obvious side effects. Of note, in the non-human primate, a cynomolgus macaque PD model, autologous iPSC derived neural cells not only elicited motor improvement but also survived 2 years after the transplantation without any immunosuppression therapy [59, 88, 94–97].

As impressive as the preclinical data is, it is still too hasty to state that clinical trials using iPSC products are ready to go. A mature transplantation protocol for PD treatment should not only address the safety issues discussed above but also refine an optimal differentiation protocol to ensure proper cell identities and long-term functions [98–100]. Hopefully, lessons and progress learned from clinical trials using iPSC products in other diseases can lend some useful experience [55, 86].

## 5. Concluding Remarks

**Figure 2:**
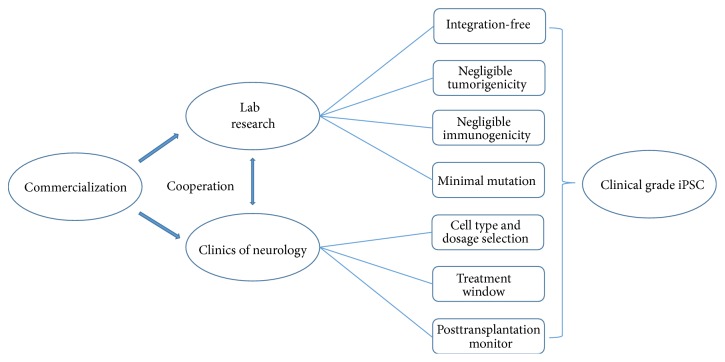
A strategy map towards clinical grade iPSCs. Strict standards should be set to ensure the safety of cell replacement therapy using iPSC products. These standards for clinical grade iPSCs must cover but are not limited to generation of iPSCs with minimal mutation, assessment for tumorigenicity and immunogenicity, cell type and dosage selection, length of treatment window, and posttranslation monitoring, which requires the cooperation of lab researchers, clinicians, and business industry.

To make human iPSCs applicable in drug screening and cell replacement therapy, aside from issues discussed above, other factors should also be taken into consideration to establish a comprehensive and multifaceted clinical standards. In some cases, therapeutists may require the use of genetically corrected syngeneic cells for transplantation; therefore, other than tumorigenicity and immunogenicity assessment related to the reprogramming process, clonal iPSC lines or iPSC derived cell lines after genome modifications should be further assayed carefully to isolate a pure line with minimal mutation to ensure safety. In addition, more efficient methods for reprogramming, colony isolation, and validation should be exploited to achieve scalable and robust biomanufacturing of iPSCs, which will not only promote the development of the drug screening industry but also facilitate collaborations across nations and laboratories. When iPSCs of consistent high quality and comparability can be generated by different institutes at desired scales flexibly, it is the time to establish a worldwide repository, where every individual could find the human leukocyte antigen (HLA) matched cells for transplantation [101]. Finally, the importance of cooperation between clinicians and researchers should not be overlooked. For the monitor markers, cell dosage for transplantation, specific surgery site, and treatment windows should be determined based on both the data from bench and the bedside medical records (Figure 2). Although lots of hurdles remain to be addressed, the good news is that researchers are not held back by these difficulties; their bold ambitions and endeavor recently yielded a guidance published by US food and drug administration, entitled “Considerations for the Design of Early-Phase Clinical Trials of Cellular and Gene Therapy Products” [102].

As a summary, undoubtedly the ultimate goal for human iPSCs studies is to provide new insight into diagnosis and therapeutics in real patients. As more knowledge is being accumulated, it is believable that commercialization of clinical grade iPSCs will come into reality in the future.

## References

[1] Takahashi K., Tanabe K., Ohnuki M. (2007). Induction of pluripotent stem cells from adult human fibroblasts by defined factors. *Cell*.

[2] Yamanaka S. (2012). Induced pluripotent stem cells: past, present, and future. *Cell Stem Cell*.

[3] Dimos J. T., Rodolfa K. T., Niakan K. K. (2008). Induced pluripotent stem cells generated from patients with ALS can be differentiated into motor neurons. *Science*.

[4] Soldner F., Hockemeyer D., Beard C. (2009). Parkinson's disease patient-derived induced pluripotent stem cells free of viral reprogramming factors. *Cell*.

[5] Yagi T., Ito D., Okada Y. (2011). Modeling familial Alzheimer's disease with induced pluripotent stem cells. *Human Molecular Genetics*.

[6] Urbach A., Bar-Nur O., Daley G. Q., Benvenisty N. (2010). Differential modeling of fragile X syndrome by human embryonic stem cells and induced pluripotent stem cells. *Cell Stem Cell*.

[7] Sebastiano V., Maeder M. L., Angstman J. F. (2011). In situ genetic correction of the sickle cell anemia mutation in human induced pluripotent stem cells using engineered zinc finger nucleases. *STEM CELLS*.

[8] Ye L., Chang J. C., Lin C., Sun X., Yu J., Kan Y. W. (2009). Induced pluripotent stem cells offer new approach to therapy in thalassemia and sickle cell anemia and option in prenatal diagnosis in genetic diseases. *Proceedings of the National Academy of Sciences of the United States of America*.

[9] Kim C., Wong J., Wen J. (2013). Studying arrhythmogenic right ventricular dysplasia with patient-specific iPSCs. *Nature*.

[10] Kamp T. J. (2011). An electrifying iPSC disease model: long QT syndrome type 2 and heart cells in a dish. *Cell Stem Cell*.

[11] Stepniewski J., Kachamakova-Trojanowska N., Ogrocki D. (2015). Induced pluripotent stem cells as a model for diabetes investigation. *Scientific Reports*.

[12] Wen Y., Chen B., Ildstad S. T. (2011). Stem cell-based strategies for the treatment of type 1 diabetes mellitus. *Expert Opinion on Biological Therapy*.

[13] Cantz T., Bleidißel M., Stehling M., Schöler H. R. (2008). In vitro differentiation of reprogrammed murine somatic cells into hepatic precursor cells. *Biological Chemistry*.

[14] Homma K., Okamoto S., Mandai M. (2013). Developing rods transplanted into the degenerating retina of Crx-knockout mice exhibit neural activity similar to native photoreceptors. *STEM CELLS*.

[15] Schwartz S. D., Hubschman J.-P., Heilwell G. (2012). Embryonic stem cell trials for macular degeneration: a preliminary report. *The Lancet*.

[16] Marchetto M. C. N., Carromeu C., Acab A. (2010). A model for neural development and treatment of rett syndrome using human induced pluripotent stem cells. *Cell*.

[17] Shi Y., Kirwan P., Smith J., MacLean G., Orkin S. H., Livesey F. J. (2012). A human stem cell model of early Alzheimer's disease pathology in Down syndrome. *Science Translational Medicine*.

[18] Peng J., Liu Q., Rao M. S., Zeng X. (2013). Using human pluripotent stem cell-derived dopaminergic neurons to evaluate candidate Parkinson's disease therapeutic agents in MPP^+^ and rotenone models. *Journal of Biomolecular Screening*.

[19] Nguyen H. N., Byers B., Cord B. (2011). LRRK2 mutant iPSC-derived DA neurons demonstrate increased susceptibility to oxidative stress. *Cell Stem Cell*.

[20] Byers B., Cord B., Nguyen H. N. (2011). SNCA triplication Parkinson's patient's iPSC-derived DA neurons accumulate *α*-synuclein and are susceptible to oxidative stress. *PLoS ONE*.

[21] Imaizumi Y., Okada Y., Akamatsu W. (2012). Mitochondrial dysfunction associated with increased oxidative stress and *α*-synuclein accumulation in PARK2 iPSC-derived neurons and postmortem brain tissue. *Molecular Brain*.

[22] Sánchez-Danés A., Richaud-Patin Y., Carballo-Carbajal I. (2012). Disease-specific phenotypes in dopamine neurons from human iPS-based models of genetic and sporadic Parkinson's disease. *The EMBO Molecular Medicine*.

[23] Israel M. A., Yuan S. H., Bardy C. (2012). Probing sporadic and familial Alzheimer's disease using induced pluripotent stem cells. *Nature*.

[24] Kondo T., Asai M., Tsukita K. (2013). Modeling Alzheimer's disease with iPSCs reveals stress phenotypes associated with intracellular A*β* and differential drug responsiveness. *Cell Stem Cell*.

[25] Woodruff G., Young J. E., Martinez F. J. (2013). The presenilin-1 DeltaE9 mutation results in reduced gamma-secretase activity, but not total loss of PS1 function, in isogenic human stem cells. *Cell Reports*.

[26] Muratore C. R., Rice H. C., Srikanth P. (2014). The familial Alzheimer's disease APPV717I mutation alters APP processing and Tau expression in iPSC-derived neurons. *Human Molecular Genetics*.

[27] Sproul A. A., Jacob S., Pre D. (2014). Characterization and molecular profiling of PSEN1 familial Alzheimer's disease iPSC-derived neural progenitors. *PLoS ONE*.

[28] Brennand K. J., Simone A., Jou J. (2011). Modelling schizophrenia using human induced pluripotent stem cells. *Nature*.

[29] Pedrosa E., Sandler V., Shah A. (2011). Development of patient-specific neurons in schizophrenia using induced pluripotent stem cells. *Journal of Neurogenetics*.

[30] Cooper O., Seo H., Andrabi S. (2012). Pharmacological rescue of mitochondrial deficits in iPSC-derived neural cells from patients with familial Parkinson's disease. *Science Translational Medicine*.

[31] Serio A., Bilican B., Barmada S. J. (2013). Astrocyte pathology and the absence of non-cell autonomy in an induced pluripotent stem cell model of TDP-43 proteinopathy. *Proceedings of the National Academy of Sciences of the United States of America*.

[32] Chae J.-I., Kim J., Lee S. G. (2012). Quantitative proteomic analysis of pregnancy-related proteins from peripheral blood mononuclear cells during pregnancy in pigs. *Animal Reproduction Science*.

[33] Guo X., Disatnik M.-H., Monbureau M., Shamloo M., Mochly-Rosen D., Qi X. (2013). Inhibition of mitochondrial fragmentation diminishes Huntington's disease-associated neurodegeneration. *The Journal of Clinical Investigation*.

[34] Eiges R., Urbach A., Malcov M. (2007). Developmental study of fragile X syndrome using human embryonic stem cells derived from preimplantation genetically diagnosed embryos. *Cell Stem Cell*.

[35] Miller J. D., Ganat Y. M., Kishinevsky S. (2013). Human iPSC-based modeling of late-onset disease via progerin-induced aging. *Cell Stem Cell*.

[36] Espuny-Camacho I., Michelsen K. A., Gall D. (2013). Pyramidal neurons derived from human pluripotent stem cells integrate efficiently into mouse brain circuits in vivo. *Neuron*.

[37] Siqueira da Fonseca S. A., Abdelmassih S., de Mello Cintra Lavagnolli T. (2009). Human immature dental pulp stem cells' contribution to developing mouse embryos: production of human/mouse preterm chimaeras. *Cell Proliferation*.

[38] Behringer R. R. (2007). Human-animal chimeras in biomedical research. *Cell Stem Cell*.

[39] Muotri A. R., Nakashima K., Toni N., Sandler V. M., Gage F. H. (2005). Development of functional human embryonic stem cell-derived neurons in mouse brain. *Proceedings of the National Academy of Sciences of the United States of America*.

[40] Maroof A. M., Keros S., Tyson J. A. (2013). Directed differentiation and functional maturation of cortical interneurons from human embryonic stem cells. *Cell Stem Cell*.

[41] Nicholas C. R., Chen J., Tang Y. (2013). Functional maturation of hPSC-derived forebrain interneurons requires an extended timeline and mimics human neural development. *Cell Stem Cell*.

[42] Cummings B. J., Uchida N., Tamaki S. J. (2005). Human neural stem cells differentiate and promote locomotor recovery in spinal cord-injured mice. *Proceedings of the National Academy of Sciences of the United States of America*.

[43] Saha K., Jaenisch R. (2009). Technical challenges in using human induced pluripotent stem cells to model disease. *Cell Stem Cell*.

[44] Yu D. X., Marchetto M. C., Gage F. H. (2013). Therapeutic translation of iPSCs for treating neurological disease. *Cell Stem Cell*.

[45] Kunze A., Giugliano M., Valero A., Renaud P. (2011). Micropatterning neural cell cultures in 3D with a multi-layered scaffold. *Biomaterials*.

[46] Kunze A., Valero A., Zosso D., Renaud P. (2011). Synergistic NGF/B27 gradients position synapses heterogeneously in 3D micropatterned neural cultures. *PLoS ONE*.

[47] Wang A., Tang Z., Park I.-H. (2011). Induced pluripotent stem cells for neural tissue engineering. *Biomaterials*.

[48] Bershteyn M., Kriegstein A. R. (2013). Cerebral organoids in a dish: progress and prospects. *Cell*.

[49] Lancaster M. A., Renner M., Martin C.-A. (2013). Cerebral organoids model human brain development and microcephaly. *Nature*.

[50] Rubin L. L. (2008). Stem cells and drug discovery: the beginning of a new era?. *Cell*.

[51] Kumari D., Swaroop M., Southall N., Huang W., Zheng W., Usdin K. (2015). High-throughput screening to identify compounds that increase fragile X mental retardation protein expression in neural stem cells differentiated from fragile X syndrome patient-derived induced pluripotent stem cells. *Stem Cells Translational Medicine*.

[52] Lee G., Ramirez C. N., Kim H. (2012). Large-scale screening using familial dysautonomia induced pluripotent stem cells identifies compounds that rescue IKBKAP expression. *Nature Biotechnology*.

[53] Ebert A. D., Yu J., Rose F. F. (2009). Induced pluripotent stem cells from a spinal muscular atrophy patient. *Nature*.

[54] Ali N., Adil S. N., Shaikh M. U. (2015). Autologous hematopoietic stem cell transplantation—10 years of data from a developing country. *Stem Cells Translational Medicine*.

[55] Alvarez Palomo A. B., McLenachan S., Chen F. K. (2015). Prospects for clinical use of reprogrammed cells for autologous treatment of macular degeneration. *Fibrogenesis & Tissue Repair*.

[56] Sasai Y. (2013). Next-generation regenerative medicine: organogenesis from stem cells in 3D culture. *Cell Stem Cell*.

[57] Oh J., Lee K., Kim H. (2015). Human-induced pluripotent stem cells generated from intervertebral disc cells improve neurologic functions in spinal cord injury. *Stem Cell Research & Therapy*.

[58] An M. C., Zhang N., Scott G. (2012). Genetic correction of Huntington's disease phenotypes in induced pluripotent stem cells. *Cell Stem Cell*.

[59] Sundberg M., Bogetofte H., Lawson T. (2013). Improved cell therapy protocols for Parkinson's disease based on differentiation efficiency and safety of hESC-, hiPSC-, and non-human primate iPSC-derived dopaminergic neurons. *STEM CELLS*.

[60] Kaji K., Norrby K., Paca A., Mileikovsky M., Mohseni P., Woltjen K. (2009). Virus-free induction of pluripotency and subsequent excision of reprogramming factors. *Nature*.

[61] Woltjen K., Michael I. P., Mohseni P. (2009). piggyBac transposition reprograms fibroblasts to induced pluripotent stem cells. *Nature*.

[62] Yu J., Hu K., Smuga-Otto K. (2009). Human induced pluripotent stem cells free of vector and transgene sequences. *Science*.

[63] Stadtfeld M., Nagaya M., Utikal J., Weir G., Hochedlinger K. (2008). Induced pluripotent stem cells generated without viral integration. *Science*.

[64] Fusaki N., Ban H., Nishiyama A., Saeki K., Hasegawa M. (2009). Efficient induction of transgene-free human pluripotent stem cells using a vector based on Sendai virus, an RNA virus that does not integrate into the host genome. *Proceedings of the Japan Academy Series B: Physical and Biological Sciences*.

[65] Kim D., Kim C.-H., Moon J.-I. (2009). Generation of human induced pluripotent stem cells by direct delivery of reprogramming proteins. *Cell Stem Cell*.

[66] Zhou H., Wu S., Joo J. Y. (2009). Generation of induced pluripotent stem cells using recombinant proteins. *Cell Stem Cell*.

[67] Gonzalez F., Monasterio M. B., Tiscornia G. (2009). Generation of mouse-induced pluripotent stem cells by transient expression of a single nonviral polycistronic vector. *Proceedings of the National Academy of Sciences of the United States of America*.

[68] Ichida J. K., Blanchard J., Lam K. (2009). A small-molecule inhibitor of Tgf-*β* signaling replaces sox2 in reprogramming by inducing nanog. *Cell Stem Cell*.

[69] Shi Y., Do J. T., Desponts C., Hahm H. S., Schöler H. R., Ding S. (2008). A combined chemical and genetic approach for the generation of induced pluripotent stem cells. *Cell Stem Cell*.

[70] Zhu G., Li Y., Zhu F. (2014). Coordination of engineered factors with TET1/2 promotes early-stage epigenetic modification during somatic cell reprogramming. *Stem Cell Reports*.

[71] Okano H., Nakamura M., Yoshida K. (2013). Steps toward safe cell therapy using induced pluripotent stem cells. *Circulation Research*.

[72] Lawrenz B., Schiller H., Willbold E., Ruediger M., Muhs A., Esser S. (2004). Highly sensitive biosafety model for stem-cell-derived grafts. *Cytotherapy*.

[73] Zhao T., Zhang Z.-N., Rong Z., Xu Y. (2011). Immunogenicity of induced pluripotent stem cells. *Nature*.

[74] Brickman J. M., Burdon T. G. (2002). Pluripotency and tumorigenicity. *Nature Genetics*.

[75] Miura K., Okada Y., Aoi T. (2009). Variation in the safety of induced pluripotent stem cell lines. *Nature Biotechnology*.

[76] Kanemura H., Go M. J., Shikamura M. (2014). Tumorigenicity studies of induced pluripotent stem cell (iPSC)-derived retinal pigment epithelium (RPE) for the treatment of age-related macular degeneration. *PLoS ONE*.

[77] Kobayashi Y., Okada Y., Itakura G. (2012). Pre-evaluated safe human iPSC-derived neural stem cells promote functional recovery after spinal cord injury in common marmoset without tumorigenicity. *PLoS ONE*.

[78] Hodges H., Pollock K., Stroemer P. (2007). Making stem cell lines suitable for transplantation. *Cell Transplantation*.

[79] Kiuru M., Boyer J. L., O'Connor T. P., Crystal R. G. (2009). Genetic control of wayward pluripotent stem cells and their progeny after transplantation. *Cell Stem Cell*.

[80] Bonini C., Bondanza A., Perna S. K. (2007). The suicide gene therapy challenge: how to improve a successful gene therapy approach. *Molecular Therapy*.

[81] Bonini C., Ferrari G., Verzeletti S. (1997). HSV-TK gene transfer into donor lymphocytes for control of allogeneic graft-versus-leukemia. *Science*.

[82] Araki R., Uda M., Hoki Y. (2013). Negligible immunogenicity of terminally differentiated cells derived from induced pluripotent or embryonic stem cells. *Nature*.

[83] Guha P., Morgan J. W., Mostoslavsky G., Rodrigues N. P., Boyd A. S. (2013). Lack of immune response to differentiated cells derived from syngeneic induced pluripotent stem cells. *Cell Stem Cell*.

[84] de Almeida P. E., Meyer E. H., Kooreman N. G. (2014). Transplanted terminally differentiated induced pluripotent stem cells are accepted by immune mechanisms similar to self-tolerance. *Nature Communications*.

[85] Zhao T., Zhang Z.-N., Westenskow P. D. (2015). Humanized mice reveal differential immunogenicity of cells derived from autologous induced pluripotent stem cells. *Cell Stem Cell*.

[86] Alper J. (2009). Geron gets green light for human trial of ES cell-derived product. *Nature Biotechnology*.

[87] Frantz S. (2012). Embryonic stem cell pioneer Geron exits field, cuts losses. *Nature Biotechnology*.

[88] Hallett P. J., Deleidi M., Astradsson A. (2015). Successful function of autologous iPSC-derived dopamine neurons following transplantation in a non-human primate model of Parkinson's disease. *Cell Stem Cell*.

[89] Jeon I., Lee N., Li J.-Y. (2012). Neuronal properties, in vivo effects, and pathology of a Huntington's disease patient-derived induced pluripotent stem cells. *STEM CELLS*.

[90] Nizzardo M., Simone C., Rizzo F. (2014). Minimally invasive transplantation of iPSC-derived ALDHhiSSCloVLA4+ neural stem cells effectively improves the phenotype of an amyotrophic lateral sclerosis model. *Human Molecular Genetics*.

[91] Simone C., Nizzardo M., Rizzo F. (2014). iPSC-derived neural stem cells act via kinase inhibition to exert neuroprotective effects in spinal muscular atrophy with respiratory distress type 1. *Stem Cell Reports*.

[92] Barker R. A., Drouin-Ouellet J., Parmar M. (2015). Cell-based therapies for Parkinson disease—past insights and future potential. *Nature Reviews Neurology*.

[93] Abbott A. (2014). Fetal-cell revival for Parkinson's. *Nature*.

[94] Wernig M., Zhao J.-P., Pruszak J. (2008). Neurons derived from reprogrammed fibroblasts functionally integrate into the fetal brain and improve symptoms of rats with Parkinson's disease. *Proceedings of the National Academy of Sciences of the United States of America*.

[95] Hargus G., Cooper O., Deleidi M. (2010). Differentiated Parkinson patient-derived induced pluripotent stem cells grow in the adult rodent brain and reduce motor asymmetry in Parkinsonian rats. *Proceedings of the National Academy of Sciences of the United States of America*.

[96] Rhee Y.-H., Ko J.-Y., Chang M.-Y. (2011). Protein-based human iPS cells efficiently generate functional dopamine neurons and can treat a rat model of Parkinson disease. *Journal of Clinical Investigation*.

[97] Emborg M. E., Liu Y., Xi J. (2013). Induced pluripotent stem cell-derived neural cells survive and mature in the nonhuman primate brain. *Cell Reports*.

[98] Arenas E., Denham M., Villaescusa J. C. (2015). How to make a midbrain dopaminergic neuron. *Development*.

[99] Chambers S. M., Fasano C. A., Papapetrou E. P., Tomishima M., Sadelain M., Studer L. (2009). Highly efficient neural conversion of human ES and iPS cells by dual inhibition of SMAD signaling. *Nature Biotechnology*.

[100] Ganat Y. M., Calder E. L., Kriks S. (2012). Identification of embryonic stem cell-derived midbrain dopaminergic neurons for engraftment. *Journal of Clinical Investigation*.

[101] Silva M., Daheron L., Hurley H. (2015). Generating iPSCs: translating cell reprogramming science into scalable and robust biomanufacturing strategies. *Cell Stem Cell*.

[102] Tabar V., Studer L. (2014). Pluripotent stem cells in regenerative medicine: challenges and recent progress. *Nature Reviews Genetics*.

[103] Hsu P. D., Lander E. S., Zhang F. (2014). Development and applications of CRISPR-Cas9 for genome engineering. *Cell*.

